# Violent Offending in Males With or Without Schizophrenia: A Role for Social Cognition?

**DOI:** 10.1093/schbul/sbad151

**Published:** 2023-10-20

**Authors:** Anja Vaskinn, Jaroslav Rokicki, Christina Bell, Natalia Tesli, Nina Bang, Gabriela Hjell, Thomas Fischer-Vieler, Unn K Haukvik, Christine Friestad

**Affiliations:** Centre for Research and Education in Forensic Psychiatry, Oslo University Hospital, Oslo, Norway; Norwegian Centre for Mental Disorders Research, Institute of Clinical Medicine, University of Oslo, Oslo, Norway; Centre for Research and Education in Forensic Psychiatry, Oslo University Hospital, Oslo, Norway; Norwegian Centre for Mental Disorders Research, Psychosis Research Section, Oslo University Hospital, Oslo, Norway; Norwegian Centre for Mental Disorders Research, Institute of Clinical Medicine, University of Oslo, Oslo, Norway; Department of Acute Psychiatry, Oslo University Hospital, Oslo, Norway; Norwegian Centre for Mental Disorders Research, Institute of Clinical Medicine, University of Oslo, Oslo, Norway; Norwegian Centre for Mental Disorders Research, Psychosis Research Section, Oslo University Hospital, Oslo, Norway; Centre for Research and Education in Forensic Psychiatry, St. Olavs Hospital, Trondheim, Norway; Department of Psychology, Norwegian University of Science and Technology, Trondheim, Norway; Department of Mental Health, Norwegian University of Science and Technology, Trondheim, Norway; Norwegian Centre for Mental Disorders Research, Institute of Clinical Medicine, University of Oslo, Oslo, Norway; Department of Psychiatry, Østfold Hospital, Grålum, Norway; Department of Clinical Research, Østfold Hospital, Grålum, Norway; Norwegian Centre for Mental Disorders Research, Institute of Clinical Medicine, University of Oslo, Oslo, Norway; Department of Mental Health and Addiction, Vestre Viken Hospital Trust, Drammen, Norway; Centre for Research and Education in Forensic Psychiatry, Oslo University Hospital, Oslo, Norway; Department of Adult Psychiatry, Institute of Clinical Medicine, University of Oslo, Oslo, Norway; Centre for Research and Education in Forensic Psychiatry, Oslo University Hospital, Oslo, Norway; University College of Norwegian Correctional Services, Lillestrøm, Norway

**Keywords:** theory of mind, emotion processing, affect perception, perspective-taking, violence, aggression

## Abstract

**Background and Hypothesis:**

Reduced social cognition has been reported in individuals who have committed interpersonal violence. It is unclear if individuals with schizophrenia and a history of violence have larger impairments than violent individuals without psychosis and non-violent individuals with schizophrenia. We examined social cognition in two groups with violent offenses, comparing their performance to non-violent individuals with schizophrenia and healthy controls.

**Study Design:**

Two social cognitive domains were assessed in four groups: men with a schizophrenia spectrum disorder with (SSD-V, *n* = 27) or without (SSD-NV, *n* = 42) a history of violence, incarcerated men serving preventive detention sentences (V, *n* = 22), and healthy male controls (HC, *n* = 76). Theory of mind (ToM) was measured with the Movie for the Assessment of Social Cognition (MASC), body emotion perception with Emotion in Biological Motion (EmoBio) test.

**Study Results:**

Kruskal–Wallis *H*-tests revealed overall group differences for social cognition. SSD-V had a global and clinically significant social cognitive impairment. V had a specific impairment, for ToM. Binary logistic regressions predicting violence category membership from social cognition and psychosis (SSD status) were conducted. The model with best fit, explaining 18%–25% of the variance, had ToM as the only predictor.

**Conclusions:**

Social cognitive impairment was present in individuals with a history of violence, with larger and more widespread impairment seen in schizophrenia. ToM predicted violence category membership, psychosis did not. The results suggest a role for social cognition in understanding interpersonal violence.

## Introduction

Interpersonal violence is a global health challenge, posing a burden to the victim, the perpetrator and the larger society.^1^ Two groups have heightened violence risk due to having committed violent interpersonal crime. One group consists of individuals with schizophrenia spectrum disorders (SSD) and a previous history of interpersonal violence. They may receive treatment at designated security facilities or specialized forensic hospitals. The other group are individuals with a history of severe interpersonal violence, but without psychosis. These individuals may serve preventive detention sentences in the regular prison system. In order to provide targeted help and individualized treatment to individuals belonging to one of these groups, a detailed account of their characteristics is of importance. An interesting avenue in that matter is their social cognitive profile, as research suggests that social cognitive impairment may be central to persons with an offender history.^2^ Reduced social cognition has been reported for violent populations^3,4^ and is present in persons with schizophrenia^5^ across geographical regions and cultural contexts.^6^

Social cognition refers to the mental processes underlying social interactions, including perceiving, interpreting, and generating responses to the intentions, dispositions, and behaviors of others.^7^ It encompasses several theoretical domains.^8^*Emotion processing* is a broad domain referring to perceiving and using emotional information, where lower-level abilities concern recognizing emotions in faces, voices or body movement. *Theory of mind* (ToM) is the ability to draw inferences about mental states. *Social perception* is defined as noticing social cues and identifying social roles and rules. *Attributional style* refers to how we explain things that happen to us.

The vast majority of community violence is not committed by individuals with schizophrenia.^9^ It is more common for a person with schizophrenia to be a victim of violence than a perpetrator, with victimization rates far exceeding rates in the general population.^10^ The overall violence risk is low, but elevated.^11^ The literature that has compared social cognition in individuals with schizophrenia with or without a history of violence has provided mixed results. There are reports of larger social cognitive impairments in persons with a history of severe interpersonal violence than those without,^12–14^ but also of the opposite.^15–17^ In some studies there are no statistically significant differences between the groups,^18,19^ at least for some of the utilized social cognitive tests,^12,15^ and significant group differences may appear on just one of two tests that assess the same domain.^12^ In addition to different characteristics of the study samples, a reason for these mixed findings could be the use of different social cognitive tests, of which some may have less than adequate psychometric properties.^20^ In sum, it is unclear if social cognitive impairment is larger in individuals with schizophrenia and a history of violence than those without.

Reduced facial affect perception is documented among prison populations,^21^ persisting in violent offenders, post-incarceration,^22^ and reduced ToM has been reported in persons convicted of sexual offenses.^23,24^ Prison populations have elevated rates of psychopathology,^25^ including psychopathy^26^ or antisocial personality disorder,^27,28^ conditions which are characterized by social cognitive impairment.^4^ Individuals with psychopathy have impairments in facial affect perception^29,30^ and appear to lack the implicit perspective-taking (ToM) that takes place in healthy people.^31^ Explicit ToM appears though, to be largely intact.^4^ Reduced ToM has been reported for antisocial personality disorder.^32^ Also, a recent meta-analysis found an association between psychopathic traits and impaired ToM.^33^ These findings suggest that social cognition may be of relevance to violence, also for individuals without schizophrenia. How the social cognitive performance level in violent individuals without psychosis compares to the level seen in persons with SSDs and a history of interpersonal violence is scarcely investigated. Krakowski et al,^34^ identified facial emotion perception deficits in individuals with schizophrenia and a history of violence, but not in a group of violent individuals without psychosis. Sedgwick et al,^35^ reviewed the literature on cognitive functions, comparing a related group, that is, those with antisocial personality disorder, to violent schizophrenia, finding that both groups had problems with facial affect perception. Whether social cognitive impairment is larger in individuals with schizophrenia and a history of violence than individuals with a history of violence, but without psychosis, is unclear.

We have three research aims. The first aim is to examine if social cognitive impairment is larger in individuals with schizophrenia and a history of violence than those without. Our second research aim is to compare the social cognitive performance of men with a history of severe interpersonal violence but with or without psychosis. Healthy male control participants serve as a reference point for presence of impairment. Our third research aim concerns the association between social cognition and violence. A prospective study of a forensic schizophrenia sample identified a direct effect of emotional processing on inpatient violence, independent of symptom load, and violence proneness.^36^ One of two identified routes to violence in schizophrenia in another study^34^ involved impaired recognition of fearful facial expressions. This suggests a role for social cognition in explaining violence perpetrated by persons with schizophrenia. Since reduced social cognition has also been reported for individuals with a history of violence without schizophrenia, it could be a general and not illness-specific risk factor for interpersonal violence. Considering the stigma and self-stigma associated with schizophrenia,^37^ it is important to examine factors that could provide nuances to the public portrayal of individuals with schizophrenia as “dangerous.” Our third research aim examines social cognition as a predictor of violence perpetration, across diagnostic groups.

## Methods

### Participants

The study was conducted at Oslo University Hospital, Østfold Hospital, Akershus University Hospital, St. Olav Hospital and at the two prison units holding prisoners serving preventive detention sentences in Norway. The study, “Violence in psychosis” (sTOP), was approved by the Regional Committee for Medical and Health Research Ethics, the Norwegian Data Protection Authority, and relevant correctional agencies. sTOP^38^ is part of the Norwegian Centre for Mental Disorders Research (NORMENT) and utilizes a similar protocol as the Thematically Organized Psychosis (TOP) umbrella research study. All participants provided written informed consent after having received information about the study.

The study comprised four groups (*n* = 167). All participants were male. The first group (V: *n* = 22) consisted of incarcerated men, serving preventive detention sentences for interpersonal violence. Interpersonal violence was defined as homicide, attempted homicide, severe violence towards other persons, and sexual offenses. In cases involving preventive detention, the accused is subject to a full psychiatric evaluation prior to sentencing, in order to establish that criteria for criminal responsibility is met. This means that the person was “non-psychotic” (according to Norwegian legal terms) and did not have a psychotic disorder. To check if the incarcerated sample might have developed a psychotic disorder after the sentence, they were reevaluated for psychotic symptoms at study inclusion, using the Positive and Negative Syndrome Scale (PANSS; see below).^39^ The second group (SSD-V: *n* = 27) had a SSD diagnosis and a history of interpersonal violence, as defined above. They were recruited from inpatient security wards at Akershus, Oslo and Østfold hospitals. Only individuals with a history of severe violence, or individuals sentenced to mandatory psychiatric treatment for a violent crime, because criteria for criminal responsibility were not met, are treated at such wards. The third group was a SSD control group without a history of violence (SSD-NV: *n* = 42) recruited from inpatient and outpatient clinics at Oslo and Akershus hospitals.^40^ Non-violence was confirmed by comprehensive information obtained from medical records and detailed interviews with the participant, further ensured by only including individuals with scores <4 (symptom absent or mild) on the PANSS G14 item (poor impulse control). Finally, healthy male participants from a previous TOP study at NORMENT,^40^ screened with the Primary Care Evaluation of Mental Disorders (PRIME-MD)^41^ interview, were included as a healthy control group (HC: *n* = 76). The HCs are randomly selected from national statistical records and invited to participation by way of letter.

Diagnostic assessments of SSD participants were conducted by trained psychologists or psychiatrists using the Structured Clinical Interview for DSM-IV,^42^ with information added from forensic reports and medical records. The DSM-IV diagnostic distribution in the SSD-V group was as follows: schizophrenia *n* = 24, schizophreniform disorder *n* = 1, other psychoses *n* = 2. In the SSD-NV group, there were 35 participants with schizophrenia and 7 with schizoaffective disorder. Exclusion criteria were age >65, head trauma leading to >10 min loss of consciousness, and somatic illness that may affect brain function. Further, to secure valid cognitive data, only participants with Norwegian as their mother tongue or who had completed their basic education in Norway were included.

### Measures

Clinical symptoms were measured with PANSS,^39^ providing scores for positive, negative, and general symptoms.

Intelligence (IQ) was assessed with the two-test version of Wechsler Abbreviated Scale of Intelligence (WASI),^43^ and social cognition was measured with two tests. The Emotion in Biological Motion (EmoBio)^44^ taps the ability to recognize four basic emotions (happiness, fear, sadness, anger) and lack of emotion (neutral) in moving bodies, that is, body emotion perception, using point-light display stimuli. The EmoBio test uses a proportional scoring method.^45^ We based our scoring on previously developed Norwegian norms.^46^ The test yields a total score, in addition to separate scores for the five emotions. ToM was indexed by the Movie for the Assessment of Social Cognition (MASC).^47^ MASC is an ecologically valid measure of the ability to ascribe feelings, thoughts and intentions to four characters in a movie. The movie depicts interactions between the four characters while they prepare dinner together. The 15-min movie is paused 45 times, and the test-taker is asked questions with a multiple-choice response format. In addition to a correct response, response options represent three error types: overmentalizing (exaggerated attribution of mental states), undermentalizing (diminished attribution of mental states), and no mentalizing errors (no attribution to mental states). Further, the questions can be divided into cognitive (26 items: MASCcog—inferring thoughts and intentions) and affective (18 items: MASCaff—inferring emotional states) ToM (see^40^ for details on allocation of items). The Norwegian version of the MASC^48^ has proven to be a valid measure, both in schizophrenia^40^ and violent populations.^24^

### Statistical Analyses

Group differences for demographic and clinical data were analyzed with univariate analyses of variance (ANOVAs). Normality checks performed with the Shapiro–Wilk test and skewness and kurtosis statistics revealed non-normality for social cognitive total scores. Therefore, group differences for social cognitive data were analyzed with non-parametric Kruskal–Wallis *H*-tests, comparing mean ranks. All four groups were included in the statistical analyses. First, Kruskal–Wallis *H*-tests were undertaken for the total scores on the EmoBio and MASC tests, respectively. In the case of statistically significant effects on these overall tests, the five EmoBio and five MASC subscores were subjected to further Kruskal–Wallis *H*-tests. Multiple testing was dealt with by applying the Benjamini–Hochberg procedure^49^ to the main analyses (EmoBio and MASC subscores, respectively), and Holm’s sequential Bonferroni procedure^50^ to post hoc group comparisons. Effect sizes were calculated for both the main (eta^2^) and post hoc (Hedge’s *g*) analyses. We used raw scores for the statistical analyses, but converted raw scores to standard scores using the mean and standard deviation of the HCs for visualization purposes. Clinical significance was also considered (−1.5 SD below HCs).^51^

As follow-up analysis, we utilized logistic regressions to test a theoretical model where social cognitive impairment contributes to interpersonal violence. “Violence” was the dependent variable. It was dichotomized into a having a history of violence (V and SSD-V) or being without a history of violence (SSD-NV and HC). Since many HCs were assessed with only one social cognitive measure, models were created with either MASC or EmoBio (total scores). Initial models included social cognition, along with “psychosis status” (presence of SSD or not) and general cognitive ability (WASI IQ) as predictors. “Psychosis status” was considered since SSD is associated with an increased risk of violence perpetration,^11^ WASI IQ because of the known relationship between social and non-social cognition^52^ and because low IQ is a risk factor for violence.^53,54^ Assumptions for logistic regression were met: there was no multicollinearity, and the Box-Tidwell procedure confirmed linearity between the continuous predictors and the logit of the dependent variable. Predictors were entered in separate blocks, starting with the main variable of interest, social cognition. Models were subsequently fitted by using the significant predictors from the initial models, and bootstrapping methods. The Statistical Package for the Social Sciences (IBM SPSS for Windows, version 28) was used for statistical analyses.

## Results

The results of the analyses can be seen in Table 1 (demographic and clinical data), 2 (EmoBio scores), 3 (MASC scores), and 4 (regression models) (Tables 2 and 3).

**Table 1.** Demographic and Clinical Information in Men With Schizophrenia Spectrum Disorder With (SSD-V) or Without (SSD-NV) a History of Interpersonal Violence, in Men Serving Preventive Detention Sentences for Interpersonal Violence (V) Compared to Healthy Male Control Participants (HC)

	SSD-V *M* (SD) *n* = 27	V *M* (SD) *n* = 22	SSD-NV *M* (SD) *n* = 42	HC *M* (SD) *n* = 76	ANOVA
Age	33.5 (8.1)	41.2 (12.9)	27.7 (8.2)	28.6 (7.0)	*F*_(3, 166)_ = 15.7, *P* < .001 SSD-V, SSD-NV, HC < V
Education (years)	11.3 (1.7) *n* = 23	13.0 (2.6) *n* = 16	12.0 (2.2)	14.5 (2.5)	*F*_(3, 156)_ = 17.3, *P* < .001 SSD-V, SSD-NV < HC
WASI IQ	93.4 (14.8) *n* = 21	100.6 (10.2) *n* = 20	100.8 (13.0)	112.3 (11.1)	*F*_(3, 158)_ = 18.5, *P* < .001 SSD-V, V, SSD-NV < HC
PANSS positive	15.0 (8.0)	7.9 (1.6) *n* = 21	14.1 (4.6)	—	*F*_(2, 89)_ = 11.9, *P*< .001 SSD-V, SSD-NV > V
PANSS negative	16.7 (7.0)	7.9 (1.1) *n* = 21	16.2 (5.0)	—	*F*_(2, 89)_ = 22.4, *P* < .001 SSD-V, SSD-NV > V
PANSS general	29.7 (8.8)	20.3 (5.0) *n* = 21	30.1 (5.3) *n* = 41	—	*F*_(2, 88)_ = 20.1, *P* < .001 SSD-V, SSD-NV > V
PANSS item G14	2.0 (1.3) *n* = 26	1.1 (0.5) *n* = 21	1.4 (0.8)	—	*F*_(2, 88)_ = 6.3, *P* = .003 SSD-V > SSD-NV, V
Antipsychotic medication DDD	1.54 (0.91) *n* = 25	—	1.39 (0.91) *n* = 37	—	*t*-test *t* = 0.63, *P* = .531

**Table 2.** Body Emotion Perception in Men With Schizophrenia Spectrum Disorder With (SSD-V) or Without (SSD-NV) a History of Interpersonal Violence, in Men Serving Preventive Detention Sentences for Interpersonal Violence (V) Compared to Healthy Male Control Participants (HC)

	SSD-V *M* (SD) *n* = 27	V *M* (SD) *n* = 21	SSD-NV *M* (SD) *n* = 42	HC *M* (SD) *n* = 45	Kruskal–Wallis *H*-tests using mean rank	Post hoc group comparisons**
EmoBio total (range 0–1)	0.73 (0.14)	0.80 (0.08)	0.81 (0.11)	0.87 (0.08)	*H*_(3)_ = 25.50 *P* < .001, η2 = 0.17	SSD-V < HC: *P* < .001, *g* = 1.32 V < HC: *P* = .010, *g* = 0.88 SSD-V < SSD-NV: *P* = .007, *g* = 0.65
EmoBio happiness (range 0–1)	0.82 (0.20)	0.89 (0.14)	0.83 (0.19)	0.89 (0.15)	*H*_(3)_ = 6.60 *P* = .086, η2 = 0.03	—
EmoBio anger (range 0–1)	0.65 (0.23)	0.73 (0.17)	0.79 (0.20)	0.79 (0.15)	*H*_(3)_ = 9.74 *P* = .021*, η2 = 0.05	SSD-V < SSD-NV: *P* = .004, *g* = 0.66
EmoBio sadness (range 0–1)	0.77 (0.21)	0.79 (0.19)	0.85 (0.19)	0.87 (0.17)	*H*_(3)_ = 8.43 *P* = .038*, η2 = 0.04	—
EmoBio fear (range 0–1)	0.63 (0.27)	0.81 (0.19)	0.73 (0.29)	0.86 (0.19)	*H*_(3)_ = 15.69 *P* < .001*, η2 = 0.10	SSD-V < HC: *P* < .001, *g* = 1.03
EmoBio neutral (range 0–1)	0.75 (0.26)	0.79 (0.20)	0.85 (0.20)	0.93 (0.12)	*H*_(3)_ = 15.87 *P* < .001*, η2 = 0.10	SSD-V < HC: *P* < .001, *g* = 0.97 V < HC: *P* = .007, *g* = 0.94

*Note*: η2 =eta squared (effect size), *g =*Hedge’s *g* (effect size).

*Significant after applying the Benjamini–Hochberg procedure.

**Only group comparisons that remained significant after Holm’s sequential Bonferroni procedure are reported.

**Table 3.** Theory of Mind in Men With Schizophrenia Spectrum Disorder With (SSD-V) or Without (SSD-NV) a History of Interpersonal Violence, in Men Serving Preventive Detention Sentences for Interpersonal Violence (V) Compared to Healthy Male Control Participants (HC)

	SSD-V *M* (SD) *n* = 25	V *M* (SD) *n* = 19	SSD-NV *M* (SD) *n* = 42	HC *M* (SD) *n* = 42	Kruskal–Wallis *H*-tests using mean rank	Post hoc group comparisons**
MASC total correct responses (range 0–45)	23.4 (8.6)	28.3 (5.1)	30.0 (6.7)	34.7 (4.3)	*H*_(3)_ = 36.07 *P* < .001, η2 = 0.27	SSD-V < HC: *P* < .001, *g* = 1.81 V < HC: *P* < .001, *g* = 1.40 SSD-NV < HC: *P* = .001, *g* = 0.85 SSD-V < SSD-NV: *P* = .004, *g* = 0.89
MASC affective ToM correct responses (range 0–18)	9.8 (3.0)	10.7 (2.8)	11.9 (2.7)	13.3 (2.1)	*H*_(3)_ = 26.57 *P* < .001*, η2 = 0.19	SSD-V < HC: *P* < .001, *g* = 1.42 V < HC: *P* = .001, *g* = 1.11 SSD-V < SSD-NV: *P* = .004, *g* = 0.75
MASC cognitive ToM correct responses (range 0–26)	13.5 (6.0)	17.3 (3.1)	17.8 (4.5)	21.1 (2.8)	*H*_(3)_ = 36.94 *P* < .001*, η2 = 0.27	SSD-V < HC: *P* < .001, *g* = 1.78 V < HC: *P* < .001, *g* = 1.31 SSD-NV < HC: *P* < .001, *g* = 0.88 SSD-V < SSD-NV: *P* = .009, *g* = 0.84
MASC overmentalizing errors (range 0–45)	5.7 (3.0)	6.1 (2.0)	5.0 (3.6)	4.7 (3.2)	*H*_(3)_ = 6.51 *P* = .089, η2 = 0.03	—
MASC undermentalizing errors (range 0–45)	10.3 (5.1)	7.0 (3.0)	6.5 (3.3)	3.9 (2.2)	*H*_(3)_ = 40.14 *P* < .001*, η2 = 0.30	SSD-V > HC: *P* < .001, *g* = 1.80 V > HC: *P* < .001, *g* = 1.26 SSD-NV > HC: *P* < .001, *g* = 0.93 SSD-V > SSD-NV: *P* = .003, *g* = 0.94
MASC no mentalizing errors (range 0–45)	5.5 (4.2)	3.7 (2.6)	3.5 (2.6)	1.7 (1.3)	*H*_(3)_ = 28.12 *P* < .001*, η2 = 0.20	SSD-V > HC: *P* < .001, *g* = 1.38 V > HC: *P* = .003, *g* = 1.11 SSD-NV > HC: *P* = .001, *g* = 0.86

*Note*: η2 =eta squared (effect size), *g =*Hedge’s *g* (effect size).

*Significant after applying the Benjamini–Hochberg procedure.

**Only group comparisons that remained significant after Holm’s sequential Bonferroni procedure are reported.

The incarcerated sample was older than the other groups and had significantly less positive and negative symptoms than the two schizophrenia groups, which did not differ significantly from each other. As expected, the HCs had higher IQ than the other groups, and longer education than participants with SSD.

Significant group differences were present for the total scores for both EmoBio and MASC, allowing us to move on to analyses of the subscores. For EmoBio, group differences were significant for all emotions, except happiness, after adjustment with the Benjamini–Hochberg procedure. SSD-V had worse performance than SSD-NV for the total score and anger, with medium effect sizes. There were no differences between SSD-V and V, but both were impaired compared to HCs. For the SSD-V group the impairment was substantial (very large effect size) and present for the total score, fear and neutral. The impairment was somewhat attenuated for the V group; seen for the total score and neutral, where it amounted to a large effect size. The non-violent SSD sample did not differ significantly from HCs on the EmoBio.

**Fig. 1.**
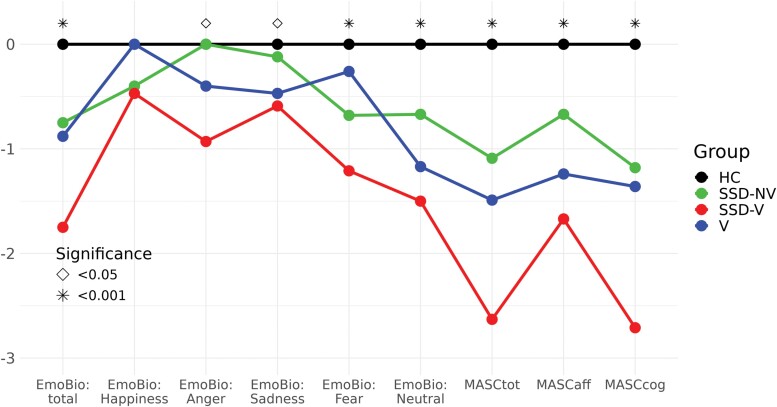
Social cognitive profile in men with schizophrenia spectrum disorder with (SSD-V) or without (SSD-NV) a history of interpersonal violence, in men serving preventive detention sentences for interpersonal violence (V) and healthy male control participants (HC). EmoBio =Emotion in Biological Motion; MASC =Movie for the Assessment of Social Cognition; MASCaff =MASC affective ToM; MASCcog =MASC cognitive ToM. Standardized scores (*z*) based on the mean and standard deviation of the healthy control group are shown.

After applying the Benjamini–Hochberg procedure, all group differences for MASC subscores, except overmentalizing errors, remained significant. For the total score, the SSD-V and V samples had significantly worse performance than HCs, with very large effect sizes. SSD-V and V did not differ significantly from each other, but the SSD-V group differed significantly from SSD-NV. The exact same pattern appeared for MASCaff, MASCcog (both correct responses), and undermentalizing errors. The three study groups (non-HCs) made significantly more no mentalizing errors than HCs, but did not differ from each other. The non-violent SSD sample was impaired compared to HCs for the total score, MASCcog, undermentalizing, and no mentalizing errors, with large effect sizes. The social cognitive performance level (standardized scores) of the four groups is available for inspection in Figure 1. (Supplementary figure 1 shows the number of MASC error types of the four groups).

The logistic regressions examining if social cognition impacted on violence category membership consistently yielded significant results. See Supplementary table 1 for details. In the initial MASC model, adding psychosis status and WASI IQ as predictors did not increase model fit, and only MASC had a unique contribution. The bootstrapped fitted model with MASC as the only predictor was statistically significant (χ^2^_(1)_ = 25.8, *P* < .001) and explained between 18 (Cox and Snell *R*^2^) and 25% (Nagelkerke *R*^2^) of the variance. Model fit of the initial EmoBio model was improved by adding WASI IQ, but not psychosis status as predictors. The bootstrapped fitted model with EmoBio and WASI IQ as predictors was statistically significant (χ^2^_(2)_ = 15.8, *P* < .001) and explained between 12 (Cox and Snell *R*^2^) and 16% (Nagelkerke *R*^2^) of the variance. WASI IQ was the only significant unique predictor. See Table 4 for fitted models.

**Table 4.** Logistic Regression Predicting Violence Category Membership

	β (S.E.)	Wald’s χ^2^	*df*	*P*	Exp(β) Odds ratio	Bootstrapped 95% CI for Exp(β)		Overall classification accuracy* (%)
						Lower	Upper	
Final fitted model MASC								
MASC total	−0.142 (0.032)	19.8	1	<.001	0.868	−0.216	−0.091	73.4
Final fitted model EmoBio								
EmoBio total	−3.200 (2.050)	2.4	1	.118	0.041	−8.962	0.833	72.4
WASI IQ	−0.041 (0.016)	6.1	1	.013	0.960	−0.077	−0.011	

*Note*:

*Percentage of cases correctly classified.

## Discussion

In this study, we identified social cognitive impairment in men with a history of interpersonal violence, regardless of the occurrence of psychosis. The magnitude and pattern of impairment, however, differed depending on whether schizophrenia was present or not. Men with schizophrenia and a history of violence had a global social cognitive impairment, present for both body emotion perception and ToM. It was clinically significant with very large effect sizes and especially pronounced for ToM. Men serving preventive detention had a more specific impairment, seen particularly for ToM and to a lesser extent for body emotion perception. For the total ToM score, their performance corresponded to a clinically significant impairment, with a very large effect size. For body emotion perception, impairment was seen for the total score, driven by reduced perception of neutral stimuli. No significant differences compared to healthy men appeared for any other emotions, suggesting a more limited impairment in emotion perception than ToM.

Our first research aim concerned whether men with schizophrenia and a history of violence performed significantly worse than men with schizophrenia without a history of violence. This was indeed the case, particularly for ToM, with large effect sizes. Prior research provided a mixed picture. The prominent differences between the violent and non-violent schizophrenia groups identified here, are, we believe, partly due to the use of well-validated tests documented to be sensitive to social cognitive impairments, both in schizophrenia^40,46,55^ and other populations.^24,56,57^ The two schizophrenia groups were recruited from slightly different settings (inpatient vs inpatient and outpatient), reflecting the treatment regime of the violent group. Institutionalization does not appear to explain the differences, as comparisons with inpatient SSD-NV participants (*n* = 5) yielded similar findings for ToM (see Supplementary material). The current results suggest that men with schizophrenia who have violently offended not only have a very poor understanding of others, but that their reduced social cognition extends beyond what we see for non-violent men with schizophrenia. For the latter group, post hoc tests identified impairments only for ToM. As can be seen in Figure 1, they scored within a standard deviation of the healthy men for almost all variables, for most closer to half a standard deviation, and did not evidence *clinically* significant impairment for any social cognitive measure. This highlights how some individuals with schizophrenia in fact present with quite limited social cognitive problems.^58^ In large part, the non-violent schizophrenia group had an intermediate performance between the healthy controls and the two groups with a history of violence, suggestive of a role of impaired social cognition in violence.

Our second research aim concerned social cognitive differences between the two violent groups. They did not differ significantly from each other, but men with schizophrenia and a history of interpersonal violence performed *numerically* worse than the incarcerated men. Different levels of social cognitive performance (see Figure 1) indicate a larger impairment in men with schizophrenia and a history of violence. Our results, although not statistically significant, align with the conclusions of a review,^35^ stating that impairments are larger in schizophrenia.

The identified ToM impairments in our incarcerated participants correspond with findings in sexual offenders^23,24^ and in antisocial personality disorder,^32^ although it must be noted that they were not recruited because they belonged to a specific violent offense or diagnostic category and therefore constitute a somewhat different group. The reduced emotion perception (from bodies), although very limited, is in line with the reduced (facial) emotion perception reported for prison populations^21,22^ and for individuals with psychopathy.^29,30^ Given the substantial impairments in ToM and quite restricted reductions in body emotion perception, our findings suggest that men with a history of violence, but without schizophrenia, have marked deficits in higher-order social cognition, that is, when making inferences about others, but to a lesser extent for low-level decoding of emotional information. In other words, their challenges in understanding the social world stem not from deficiencies in the rapid recognition of emotions, but involve making deliberate guesses about the internal state of others. Taken together, our results point to ToM as most important when attempting to understand violence.

Our logistic regression analyses also supported this notion. Both regression models were significant. In our fitted ToM model, ToM was the only predictor of violence category membership. The fitted emotion perception model included emotion perception and IQ as predictors of violence category membership, with only IQ having a unique contribution. The ToM model explained more of the variance than the emotion perception model, aligning with the results of our group comparisons. Importantly, “psychosis status,” that is, having a SSD diagnosis, was not associated with belonging to the violent category in either model. This indicates that having a SSD diagnosis might not be the most decisive feature for interpersonal violence in individuals presenting with schizophrenia, but rather their degree of social cognitive impairment. This has important consequences for anti-stigma work. Although schizophrenia is associated with increased violence risk with up to 5% of women and 25% of men committing violent acts,^11^ most individuals with the diagnosis are not violent. One hypothesis emerging from our work is that it is not schizophrenia per se, but impairment in higher-order social cognition that contributes to the elevated rates of violence in this population. Specifically, it appears that undermentalizing is essential. Overall group differences did not appear for overmentalizing errors on the MASC test. Consequently, reduced ToM in the two groups with a history of interpersonal violence cannot be due to an exaggerated interpretation of others’ intentions and emotions. Rather, undermentalizing, that is, failing to pick up all relevant information, seems to be at the core of the ToM impairment identified in this study. The male perpetrators were able to attribute mental states, evidenced by making few “no mentalizing” errors, significantly fewer than other errors (see Supplementary material). However, something appears to go awry during the reading of the other’s mind, in that important social information gets lost. A failure to understand the richness of social situations could be of importance for committing interpersonal violence. Whether undermentalizing stands in the way of empathy emerging,^59^ or perhaps increases the chances of misunderstandings thereby eliciting aggression, remain speculations.

The study has several limitations. Although our results suggest a role of impaired social cognition in violence, the cross-sectional design means we cannot know if this impairment was present before the violent act. The impact of other features at the time of offense, such as use of substances, known to increase the risk of perpetrating violence^11^ is also unknown. We cannot rule out that non-psychotic mental illness may have affected social cognition in the incarcerated men as they did not undergo diagnostic assessments. Further, we did not examine the influence of psychopathy since such data was not collected for our non-violent study samples. We expect that psychopathy to some extent explains violence category membership. However, in a previous investigation using largely overlapping samples, most of the individuals in the groups with a history of violence scored below psychopathy cutoff values,^60^ suggesting that we need to look beyond psychopathy to identify predictors of interpersonal violence. Environmental factors, such as trauma history, may be associated with both impaired social cognition^61^ and violence,^62^ but was not examined. Further, we only assessed two social cognitive domains, but acknowledge that social cognitive bias^63^ such as hostile attributional style^64^ may also play a role in violence. Finally, how well the samples represent the larger population they belong to, is unknown, and the study only included males. Broad generalizations should be avoided.

In sum, this study found that social cognitive impairment is present in men with a history of violence, with or without SSDs. The two groups did not differ significantly from each other, but men with schizophrenia performed below non-violent schizophrenia participants. Their impairment was generalized and substantial. For the incarcerated sample, the social cognitive impairment was pronounced for ToM, and of a clinically significant degree. Logistic regressions supported the initial findings, pointing to ToM as a feature possibly underlying interpersonal violence. Our study supports the notion that social cognitive impairment contributes to interpersonal violence, at least when defined as belonging to a category with a history of violent offending.

Implications of the study are that social cognitive interventions should be considered for individuals with a history of violence, regardless of whether they receive treatment in hospitals or are detained in prisons. Studies in SSD populations indicate that social cognition training can reduce violence and aggression, both in thinking and behavior.^65^ We see potential for violence prevention through social cognition training in vulnerable groups. Furthermore, increasing the knowledge^66^ of the role of social cognition in violence has the potential to reduce stigma. This has consequences for those living with schizophrenia, policy makers and the public.

## Supplementary Material

sbad151_suppl_Supplementary_Figures_1_Tables_1
