# Supplementary material for: Violent Offending in Males With or Without Schizophrenia: A Role for Social Cognition?
Source: Schizophr Bull. 2023 Oct 20;50(3):663–72. doi: 10.1093/schbul/sbad151 (PMC11059786; doi:10.1093/schbul/sbad151)
Supplement: sbad151_suppl_Supplementary_Figures_1_Tables_1 [file sbad151_suppl_supplementary_figures_1_tables_1.docx]

**Suppl. Figure 1.** Theory of mind (MASC): number of errors in men with schizophrenia spectrum disorder with (SSD-V) or without (SSD-NV) a history of interpersonal violence, in men serving preventive detention sentences for interpersonal violence (V) and healthy male control participants (HC)

**Suppl. Table 1.** Logistic regression predicting violence category membership.

|  |  |  |  |  |  |  | 95% CI for Exp(*β*) | |  |
| --- | --- | --- | --- | --- | --- | --- | --- | --- | --- |
|  | Overall model fit | *β* (S.E.) | Wald’s χ^2^ | df | p | Exp(*β*)  Odds ratio | Lower | Upper | Overall classification accuracy* |
| ***Initial model MASC*** |  |  |  |  |  |  |  |  |  |
| Block 1:  MASC total | χ^2^ _(1)_ = 19.6, p < 0.001 |  |  |  |  |  |  |  | 74.0 % |
|  |  | -0.128 (0.032) | 16.1 | 1 | < 0.001 | 0.879 | 0.826 | 0.936 |  |
| Block 2:  MASC total  Psychosis status | χ^2^ _(2)_ = 21.6, p < 0.001 |  |  |  |  |  |  |  | 75.6 % |
|  |  | -0.144 (0.035) | 17.0 | 1 | < 0.001 | 0.866 | 0.809 | 0.927 |  |
|  |  | 0.591 (0.463) | 1.6 | 1 | 0.202 | 1.806 | 0.728 | 4.477 |  |
| Block 3:  MASC total  Psychosis status  WASI IQ | χ^2^ _(3)_ = 24.0, p < 0.001 |  |  |  |  |  |  |  | 72.4 % |
|  |  | -0.119 (0.038) | 9.8 | 1 | 0.002 | 0.888 | 0.825 | 0.957 |  |
|  |  | 0.733 (0.480) | 2.3 | 1 | 0.126 | 2.081 | 0.813 | 5.328 |  |
|  |  | -0.028 (0.019) | 2.3 | 1 | 0.128 | 0.972 | 0.937 | 1.008 |  |
| ***Initial model EmoBio*** |  |  |  |  |  |  |  |  |  |
| Block 1:  EmoBio total | χ^2^ _(1)_ = 9.4, p = 0.002 |  |  |  |  |  |  |  | 69.3 % |
|  |  | -5.302 (1.850) | 8.2 | 1 | 0.004 | 0.005 | 0.000 | 0.187 |  |
| Block 2:  EmoBio total  Psychosis status | χ^2^ _(2)_ = 9.5, p = 0.009 |  |  |  |  |  |  |  | 69.3 % |
|  |  | -5.485 (1.922) | 8.1 | 1 | 0.004 | 0.004 | 0.000 | 0.180 |  |
|  |  | 0.152 (0.412) | 0.1 | 1 | 0.712 | 1.165 | 0.519 | 2.613 |  |
| Block 3:  EmoBio total  Psychosis status  WASI IQ | χ^2^ _(3)_ = 17.3, p < 0.001 |  |  |  |  |  |  |  | 74.0 % |
|  |  | -3.497 (2.088) | 2.8 | 1 | 0.094 | 0.030 | 0.001 | 1.815 |  |
|  |  | 0.539 (0.450) | 1.4 | 1 | 0.231 | 1.714 | 0.710 | 4.139 |  |
|  |  | -0.047 (0.017) | 7.3 | 1 | 0.007 | 0.954 | 0.922 | 0.987 |  |

*Percentage of cases correctly classified

**Suppl. Figure 2.** Boxplot of MASC total score in the four study groups


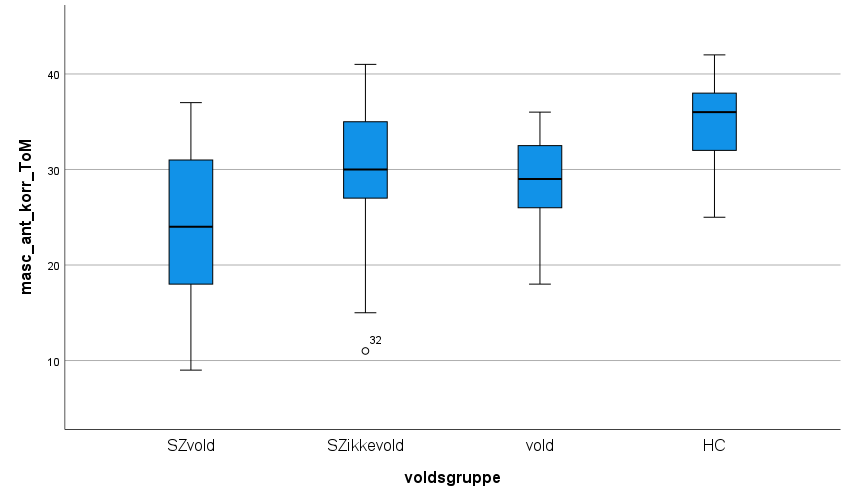


**Suppl. Figure 3.** Boxplot of EmoBio total score in the four study groups


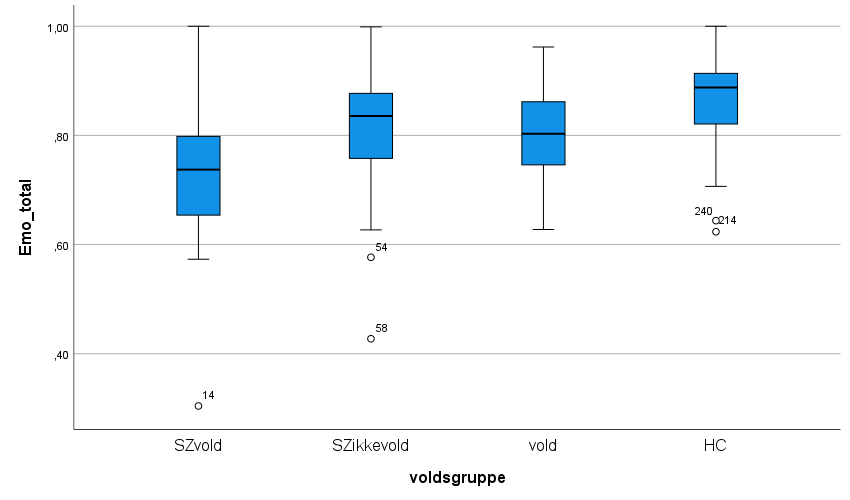


**Statistical comparison of social cognition in SSD-V participants with performance of SSD-NV participants who received inpatient treatment (n = 5) (referred to in the Discussion).**

Independent samples *t*-tests were undertaken. Please note that data concerning inpatient/outpatient status is missing for 11 SSD-NV participants, i.e. 26% of the sample. We do not know if the five individuals in the SSD-NV group included in these analyses are representative for the inpatient subsample of the entire SSD-NV sample.

For EmoBio total (SSD-V n = 27), the difference was small (*t* = 0.07, p = 0.943, Cohen’s *d* effect size = 0.04). For MASCtotal (SSD-V n = 25), the difference was large with an effect size of Cohen’s *d* = 0.63 (*t* = 1.29, p = 0.208). The differences were not statistically significant. See table for numbers and results:

**Suppl. Table 2.** Group comparisons of social cognition, only participants with inpatient status included in the SSD-NV group.

|  | SSD-NV | SSD-NV inpatient (n = 5) | Statistics | Cohen’s *d* |
| --- | --- | --- | --- | --- |
| EmoBio total | 0.73 (0.14) | 0.75 (0.19) | *t* = 0.07, p = 0.943 | 0.04 |
| MASC total | 23.4 (8.6) | 28.9 (8.2) | *t* = 1.29, p = 0.208 | 0.63 |

**Statistical analyses of differences in the number of ToM errors (on the MASC test) among male perpetrators (referred to in the Discussion).**

We conducted within-group analyses of MASC error types in male perpetrators (SSD-V and V samples, combined). Since data was non-normally distributed, two Related-Samples Wilcoxon Signed Rank Tests were done. The number of ‘no mentalizing errors’ differed significantly from the number of ‘undermentalizing errors’ (z = 5.0, p < 0.001) as well as from the number of ‘overmentalizing errors’ (z = 2.2, p = 0.029).
